# *PPARA* genetic variants increase the risk for cardiac pumping function reductions following acute high‐altitude exposure: A self‐controlled study

**DOI:** 10.1002/mgg3.919

**Published:** 2019-08-12

**Authors:** Jie Yang, Chuan Liu, Zhang Jihang, Jie Yu, Limeng Dai, Xiaohan Ding, Youzhu Qiu, Sanjiu Yu, Yuanqi Yang, Yuzhang Wu, Lan Huang

**Affiliations:** ^1^ Department of Cardiology the Second Affiliated Hospital, Army Medical University (Third Military Medical University) Chongqing PR China; ^2^ Department of Medical Genetics, College of Basic Medical Science Army Medical University (Third Military Medical University) Chongqing PR China; ^3^ Institute of Immunology Army Medical University (Third Military Medical University) Chongqing PR China

**Keywords:** cardiac function, genotype–phenotype correlation, haplotypes, high altitude, *PPARA*

## Abstract

**Background:**

Left cardiac pumping function determines the compensatory capacity of the cardiovascular system following acute high‐altitude exposure. Variations in cardiac output (CO) at high altitude are inconsistent between individuals, and genetic susceptibility may play a crucial role. We sought to identify genetic causes of cardiac pumping function variations and describe the genotype–phenotype correlations.

**Methods:**

A total of 151 young male volunteers were recruited and transferred to Lhasa (3,700 m) from Chengdu (<500 m) by plane. Genetic information related to hypoxic signaling and cardiovascular‐related pathways was collected before departure. Echocardiography was performed both before departure and 24 hr after arrival at high altitude.

**Results:**

Here we reported that *PPARA* variants were closely related to high‐altitude cardiac function. The variants of rs6520015 C‐allele and rs7292407 A‐allele significantly increased the risk for cardiac pumping function reductions following acute high‐altitude exposure. In addition, the individuals carrying haplotypes in *PPARA*, namely, rs135538 C‐allele, rs4253623 A‐allele, rs6520015 C‐allele and rs7292407 A‐allele (C‐A‐C‐A), suffered a 7.27‐fold risk for cardiac pumping function reduction (95% CI: 2.39–22.15, *p *=* *.0006) compared with those carrying the wild‐type haplotype.

**Conclusions:**

This self‐controlled study revealed that *PPARA* variations significantly increased the risk for cardiac pumping function reductions following acute high‐altitude exposure, providing a potential predictive marker before high‐altitude exposure and targets in mechanistic studies.

## 1 INTRODUCTION

High‐altitude hypobaric hypoxia represents a major physiological stressor and arouses a series of compensatory responses in the body to adapt to changing circumstance (Zarndt, Walls, Ocorr, & Bodmer, 2017). The circulatory and respiratory systems play primary roles in the progression of acclimatization following acute hypoxia exposure (West, 1982). Effective compensation of the two systems facilitates acclimatization to acute hypoxia and avoids the occurrence of high‐altitude‐associated symptoms (Maufrais et al., 2017; Naeije, 2010).

In the respiratory system, hypoxia induces a peripheral chemoreceptor afferent activity rise and increases ventilation and sympathetic activity (Dempsey & Smith, 2014). Pulmonary vasoconstriction and sympathetic activation further lead to pulmonary hypertension (Bartsch & Gibbs, 2007; Parati et al., 2018). On the other hand, the consequences of acute hypoxia are an increase in ejection fraction (EF) and cardiac output (CO) as a result of improvements in myocardial contractility and heart rate (HR) (Bartsch & Gibbs, 2007; Higgins, Tuttle, & Higgins, 2010). However, not all individuals at high altitude exhibit improvements in CO during the compensatory progression of the circulatory system. Some reports indicate that high‐altitude exposure can maintain an unchanged or a slightly reduced CO due to the shortening of the isovolumetric relaxation time (IVRT) and impairment of left ventricular (LV) filling that are attributed to the HR increase and elevation in pulmonary vascular resistance secondary to hypoxic pulmonary vasoconstriction (Boussuges et al., 2000; Maufrais et al., 2017; Stembridge et al., 2019, 2015; Stickland, 2018). At the same time, the plasma volume decreases with acclimatization to high altitude, and hypovolemia contributes to the decline in stroke volume (SV) (Stembridge et al., 2019). Thus, variations in CO in individuals after acute high‐altitude exposure are inconsistent, and these variations may determine the cardiac compensatory capacity.

A previous study revealed that highland dwellers (Sherpa) exhibited slower LV diastolic relaxation and a lower peak LV untwisting velocity than lowland controls (Caucasian) (Stembridge et al., 2016). In addition, Murray *et al*. demonstrated a lower capacity for fatty acid oxidation (FAO) in the skeletal muscles of the Sherpa (Murray, Montgomery, Feelisch, Grocott, & Martin, 2018). The peroxisome proliferator‐activated receptor A (*PPARA, OMIM:* *170998*) gene, which is associated with FAO, has been further confirmed to be enriched in highland natives (Horscroft et al., 2017; Simonson et al., 2010). FAO serves as the major source of energy for cardiomyocytes in healthy individuals. The lower capacity of FAO is beneficial to the cardiac function compensation and adaptation of the Sherpa. These results strongly suggest that genetic differences in myocardial metabolism may determine the variations in cardiac function and compensatory capacity at high altitude.

However, until now, the genetic basis of cardiac function variations after acute high‐altitude exposure has been poorly understood. Thus, in this self‐controlled study, we analyzed 42 polymorphisms in hypoxia‐inducible factor (HIF)‐related (*HIF1A, OMIM: 603348*, *EPAS1, OMIM: 603349*, *EGLN1, OMIM: 606425*, *EGLN3, OMIM: 606426*, *EPAS1, OMIM: 603349*, *HMOX2, OMIM: 141250*, *HIF1AN, OMIM: 606615*) and cardiovascular regulatory‐related pathways (*PPARA, OMIM: 170998*, *VEGFA, OMIM: 192240*, *ACE, OMIM: 106180*, *NOS3, OMIM: 163729*, *EDN1, OMIM: 131240*, *ANGPTL4, OMIM: 605910*, *AGT, OMIM: 106150*). We aimed to evaluate their associations with cardiac function variations in acute high‐altitude exposure. Furthermore, we planned to explore the potential haplotype and predict the individuals who most likely suffered cardiac pumping function reductions at high altitude.

## 2 MATERIALS AND METHODS

### 2.1 Ethical compliance

This study was registered in the Center of Chinese Clinical Trial Registration (NO: ChiCTR‐RCS‐12002232), and all procedures were approved by the Clinical Research Ethics Board at the Third Military Medical University (Army Medical University) (identification code: 2012014 approved on May 9, 2012) and conformed to the standards set by the Declaration of Helsinki.

### 2.2 Study population

**Figure 1:**
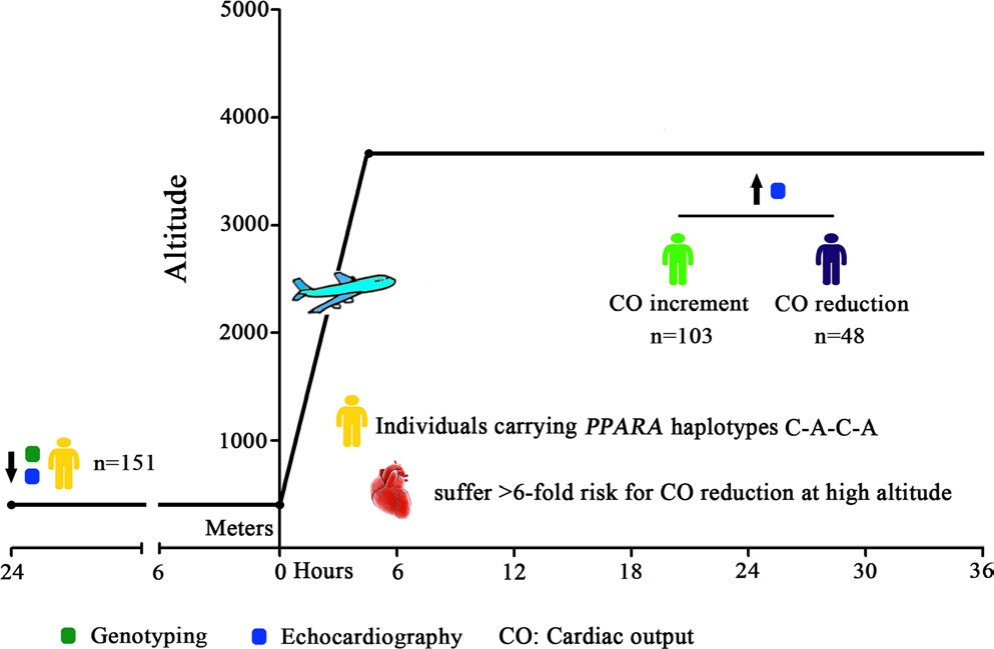
Ascending process. All the subjects were carried to Lhasa (High altitude, HA, 3,700 m, above sea level, asl) from Chengdu (sea level, LA, <500 m) by plane. The echocardiography was performed both at LA and 24 hr after arrival at HA as the blue circle marked. The demographic data as well as the gene information were obtained at LA before departure as the green circle marked

The present prospective self‐controlled study was performed from June to July of 2012. A total of 151 young healthy lowland males were recruited according to our exclusion criteria at Chengdu (low altitude, LA, 400 m**)**, following written informed consent as approved by the local ethics committee. Exclusion criteria included the following: (a) cardiovascular or respiratory disease; (b) incomplete or abnormal echocardiogram; (c) previous residence in a plateau area; and (d) other serious illnesses. To avoid the confounding effects of sex, all participants were Chinese Han men, aged 17 to 41 years, whose genetic information was extracted from peripheral blood. All participants were transferred to Lhasa (3,400 m) by plane for approximately 2 hr (Figure 1).

### 2.3 Echocardiography

The echocardiographic study was performed with an ultrasound system (CX 50, Philips Ultrasound System, Andover, MA, USA); with a probe of 2 to 4 MHz that was used to evaluate the left cardiac function both at sea level and at high altitude. The left atrial and ventricular diameters and the thicknesses of the interventricular septum (IVS) and left ventricular posterior wall (LVPW) were measured via ultrasonogram. The left ventricular end‐systolic volume (LVESV) and left ventricular end‐diastolic volume (LVEDV) were calculated according to the "method of discs" to derive the left ventricular ejection fraction (LVEF) and SV (Schiller et al., 1989). CO was calculated as SV multiplied by HR. To avoid interference by physical activity, data collection was carried out at least 30 min after rest. All reported measurements were on behalf of the average of three consecutive cardiac cycles. The echocardiography was carried out by same well‐trained operators who adopted strict reading criteria and were blinded to the information of the subjects.

### 2.4 SNP selection and genotyping

Altogether, 42 putative SNPs in *HIF1A*, *EPAS1*, *EGLN1*, *EGLN3*, *EPAS1*, *HMOX2*, *HIF1AN, PPARA*, *VEGFA*, *ACE*, *NOS3*, *EDN1*, *ANGPTL4,* and *AGT* were selected from the dbSNP database (http://www.ncbi.nlm.nih.gov/SNP) and the genotyping data of the HapMap project (http://www.1000genomes.org/) for analysis. Each of the SNPs had minor allele frequencies (MAFs) >5% in the Chinese Han population. Genomic DNA was extracted from whole blood in accordance with the instructions provided with the AxyPrep Blood Genomic DNA Miniprep Kit (Axygen Biosciences, Union City, CA, USA). Genomic DNA samples were stored at −20°C until analysis. PCR primer pairs were designed using Sequenom Assay Design software (version 3.1, Sequenom Inc., San Diego, CA, USA) and synthesized by Sangon Biotech. Genotyping was performed using the MassARRAY MALDI‐TOF platform (Sequenom Inc., San Diego, CA, USA) in accordance with the manufacturer's protocol. Sequenom Typer 4.0 software was used to perform data management and analysis.

### 2.5 Covariates

The demographic parameters, namely, age, height, weight, and drinking and smoking history, were recorded. Physiological characteristics such as systolic blood pressure (SBP) and diastolic blood pressure (DBP) were measured with an Omron HEM‐6200 (Japan). Oxygen saturation (SpO_2_) was detected with warmed hands at the fingertip with a pulse oximeter (Nonin ONYX OR9500, USA). All measurements were performed after the participants rested for more than 30 min and were repeated more than twice.

### 2.6 Statistical analysis

The Kolmogorov–Smirnov test was used to check for a normal distribution of continuous data. Normally distributed continuous data were expressed as the mean ± standard deviation. Comparisons between groups of normal distribution were made with the independent‐samples *t* test. A paired‐samples *t* test was employed to compare self‐matching data. The median (interquartile range) was used to represent the data that did not meet the criteria for a normal distribution. Comparisons between groups and self‐matching data of abnormal distribution were made with Mann–Whitney and Wilcoxon signed‐rank tests, respectively. A chi‐square test was employed to compare categorical variables between groups. The SPSS version 19.0 statistical package (SPSS, Chicago, IL, USA) was employed for statistical analysis. A *p*‐value of <.05 was considered statistically significant. *p* values for multiple testing were corrected according to Bonferroni–Holm method and *p* values of <.001 were considered statistically significant. Genotype and allele distributions, OR (odds ratio) and 95% CI (confidence interval) were calculated by multivariate logistic regression. The covariates used were age, height, weight, and drinking and smoking history. SNPSatas (https://www.snpstats.net/start.htm) online software was used to confirm Hardy–Weinberg equilibrium and allele frequencies. We used the Haploview software package (version 4.2) to analyze the linkage disequilibrium (LD) and haplotypes.

## 3 RESULTS

### 3.1 Demographic and physiological parameters of the study cohort

A total of 151 subjects were studied, and the characteristics of age, height, weight, smoking and drinking history are summarized in Table 1. Oxygen saturation (SpO_2_, high altitude [HA] 89 [87–91] vs. low altitude [LA] 98 [98–99] %, *p *<* *.001) decreased, but DBP (HA 77 ± 10 vs. LA 74 ± 9 mmHg, *p *=* *.005) and mean arterial pressure (MAP, HA 91 ± 9 vs. LA 88 ± 9 mmHg, *p *=* *.028) increased following acute high‐altitude exposure for 24 hr. No significant difference in SBP was observed, as shown in Table 1.

**Table 1:** Demographic and physiological parameters of study cohort

	LA (*n* = 151)	HA (*n* = 151)	*p* value
Demographic parameters			
Age	22 (20–25)		
Height (cm)	171 (168–175)		
Weight (kg)	64 (60–68)		
BMI	22 (20–23)		
Smoking history			
Yes (*n*, %)	112 (74)		
No (*n*, %)	39 (26)		
Drinking history			
Yes (*n*, %)	94 (62)		
No (*n*, %)	57 (38)		
Physiological characters			
SpO2 (%)	98 (98–99)	89 (87–91)	<.001
SBP (mmHg)	117 ± 11	117 ± 12	.787
DBP (mmHg)	74 ± 9	77 ± 10	.005
MAP (mmHg)	88 ± 9	91 ± 9	.028

Note

Values are presented as mean ± standard deviation or median (range interquartile); *p *<* *.05 indicate statistical significance.

Abbreviations: LA, low altitude; HA, high altitude; BMI, body mass index; SBP, systolic blood pressure; DBP, diastolic blood pressure; MAP, mean arterial pressure; SpO2, oxygen saturation.

### 3.2 Echocardiographic parameters of the study cohort at sea level and high altitude

Echocardiographic parameters of the study cohort listed in Table 2. The diameter of LV and the LVEDV and LVESV showed no prominent deviations following acute high‐altitude exposure for 24 hr. However, the diameter of LA decreased slightly (HA 30.0 [29.0–31.0] vs. LA 30.3 [29.0–31.7] mm, *p *=* *.018). Both the IVS and the LVPW decreased remarkably, and LVEF improved from 64.0 (60.0–68.0) to 66.3 (64.0–70.0) % (*p *<* *.001), which indicated that acute hypoxia exposure enhanced the contractility of the ventricular myocardium to elevate the ejection. Additionally, HR increased prominently from 67 (60–75) to 80 (72–88) beats/min (*p *<* *.001) with SV slightly increasing, which led to a pronounced rise in CO (HA 5.1 [4.3–5.8] vs. LA 4.4 ± 1.0 L/min, *p *<* *.001) after acute hypoxia exposure. CO has been confirmed and widely utilized to represent cardiac pumping function among individuals (King & Lowery, 2019). CO determines the oxygen delivery (oxygen delivery [DO_2_] = CO × SpO_2_) and compensatory capacity (Picod, Blanchard, & Cohen, 2018). Therefore, these data suggest that acute hypoxia increases the left cardiac pumping function to compensate for SpO_2_ insufficiency at high altitude.

**Table 2:** Echocardiographic parameters at low altitude and high altitude

Echocardiography			
	LA (*n* = 151)	HA (*n* = 151)	*p* value
LA (mm)	30.3 (29.0–31.7)	30.0 (29.0–31.0)	.018
LV (mm)	46.9 (44.0–48.0)	46.5 (44.6–48.0)	.310
IVS (mm)	10.6 (9.9–11.0)	10.0 (9.4–10.5)	<.001
LVPW (mm)	10.0 (9.3–10.3)	9.3 (8.7–10.0)	<.001
LVEF (%)	64.0 (60.0–68.0)	66.3 (64.0–70.0)	<.001
LVEDV (ml)	103.0 (95.0–112.0)	102.9 ± 15.9	.481
LVESV (ml)	39.0 ± 8.9	38.7 ± 10.2	.826
SV (ml)	63.0 (57.8–71.0)	64.1 ± 13.6	.609
HR (beats/min)	67 (60–75)	80 (72–88)	<.001
CO (L/min)	4.4 ± 1.0	5.1 (4.3–5.8)	<.001

Note

Values are presented as mean ± standard deviation or median (range interquartile); *p* < .05 indicate statistical significance.

Abbreviations: LA, low altitude; HA, high altitude; LA, left atrial diameter; LV, left ventricular diameter; IVS, interventricular septum; LVPW, left ventricular posterior wall; LVEF, left ventricular ejection fraction; LVEDV, left ventricular end‐diastolic volume; LVESV, left ventricular end‐systolic volume; SV, stroke volume; HR, heart rate; CO, cardiac output.

Although the results of our cohort exhibited an improvement in left cardiac pumping function in general. Forty‐eight (31.79%) subjects still suffered left cardiac pumping function reductions in our study, and their COs decreased at high altitude in comparison to sea level. According to the variations in CO, we divided the cohort into the CO improvement group (COI, *n* = 103) and the CO reduction group (COR, *n* = 48). Two groups showed no marked deviations in demographic or physiological parameters, including age, weight, height, BMI, SpO_2_, SBP, DBP, MAP or smoking and drinking history at sea level (Supplemental Table S1).

### 3.3 SNP information and genotypic frequencies

Previous studies have shown different left cardiac function changes between highland natives and lowlanders after acute hypoxia exposure (Maufrais et al., 2017; Naeije, 2010; Stembridge et al., 2019, 2016). Therefore, we considered that genetic background might determine the left cardiac pumping function of individuals. To analyze the effect of genetic factors on cardiac pumping function changes in acute high‐altitude exposure, we obtained DNA from all individuals and identified 42 SNPs. The genotypes, MAF and Hardy–Weinberg equilibrium of the SNPs in *PPARA* were shown in Table 3 and Table S2. No significant deviations from Hardy–Weinberg equilibrium were observed for any of the genetic variants.

**Table 3:** *PPARA* SNPs information and genotypic frequencies of the study cohort

*PPARA* SNP numbers	Genotypes	Frequency cases (*n*, %)	MAF	*P*‐HWE
rs135538	G/G	59 (39)	0.404	.090
	C/G	62 (41)		
	C/C	30 (20)		
rs4253623	A/A	105 (70)	0.172	.390
	A/G	40 (26)		
	G/G	6 (4)		
rs4253681	T/T	110 (73)	0.139	.310
	C/T	40 (26)		
	C/C	1 (1)		
rs4253747	T/T	114 (76)	0.126	.470
	A/T	36 (23)		
	A/A	1 (1)		
rs6520015	T/T	107 (71)	0.162	.550
	C/T	39 (26)		
	C/C	5 (3)		
rs7292407	C/C	110 (73)	0.149	.750
	A/C	37 (24)		
	A/A	4 (3)		

Abbreviations: SNP, single nucleotide polymorphism; HWE, Hardy–Weinberg Equilibrium; MAF, minor allele frequency.

### 3.4 Correlation between genotypes and cardiac function variations

**Figure 2:**
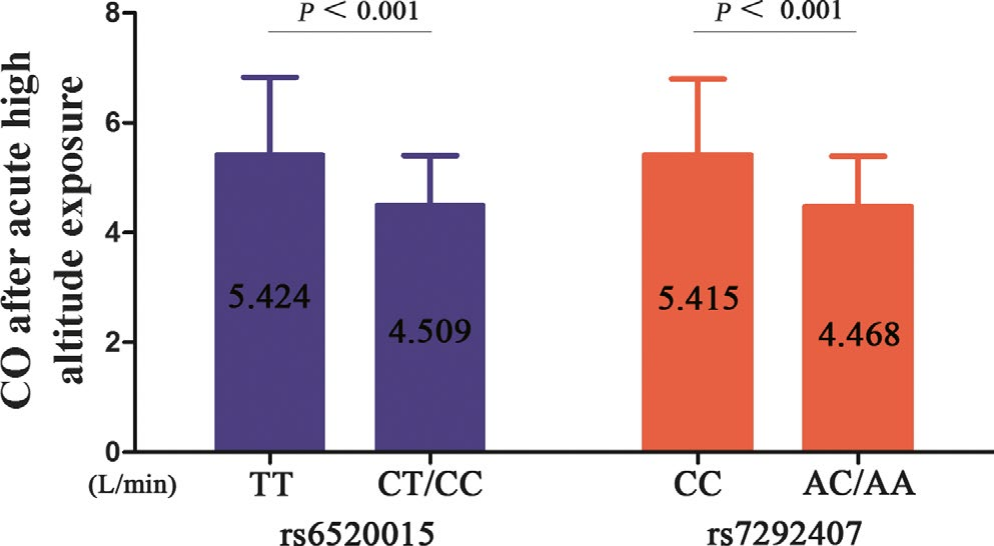
CO after acute high‐altitude exposure in alteration and wide‐type groups. Subjects were divided into wild and alteration groups in the two SNPs of *PPARA*. Both the alteration of the SNPs showed lower CO in comparison to the wide types. The CO was 5.424 ± 1.804 L/min in the subjects of carrying TT genotype and 4.509 ± 1.110 L/min in the subjects carrying CT and CC genotype in rs6520015 (*p *<* *.001)；The CO of CC genotype in rs7292407 was 5.415 ± 1.893 L/min related to alteration group of 4.468 ± 1.088 L/min (*p *<* *.001); *p *<* *.001 indicate statistical significance

We assessed the associations between these SNPs and cardiac pumping function changes in different models following acute high‐altitude exposure. The data are shown in Supplemental Table S3. Among these SNPs, logistic regression analysis revealed that the *PPARA* gene (rs6520015 and rs7292407) was associated with cardiac pumping function reductions in acute high‐altitude exposure. The rs6520015 C‐allele and rs7292407 A‐allele variations increased the risk for cardiac pumping function reductions in the univariate analysis, with ORs of 4.73 (95% CI: 2.26–10.21, *p *<* *.001) and 5.06 (95% CI: 2.35–10.91, *p *<* *.001, Table 4), respectively. This risk was more significant after adjusting for confounders with OR_adj_ of 5.26 (95% CI: 2.37–12.11, *p *<* *.001) and 5.95 (95% CI: 2.61–13.58, *p *<* *.001, Table 4). Individuals homozygous for the wild‐type allele were compared to subjects carrying the variant. The CO of subjects carrying the rs6520015 C‐allele was reduced to 4.509 ± 0.894 L/min in comparison to those carrying the individuals homozygous for the wild‐type allele, with a CO of 5.424 ± 1.403 L/min (*p *<* *.001), at high altitude. Likewise, carriers of the rs7292407 A‐allele exhibited a lower value of CO (4.468 ± 0.921 L/min) than the individuals homozygous for the wild‐type allele (5.415 ± 1.383 L/min) (*p *<* *.001) (Figure 2). In addition, no significant differences were found in the correlation analysis with the CO after acute high‐altitude exposure among *EGLN1* (rs12406290, rs12757362, rs1339891, rs1339894, rs1361384, rs1538667, rs2009873, rs2066140, rs2153364, rs2275279, rs2486729, rs2739513, rs2808609, rs50508618), *HIF1AN* (rs10883512, rs2295778), *HIF1A* (rs11549467, rs12434438, rs2301113), *PPARA* (rs4253623, rs4253681, rs4253747), *VEGFA* (rs10434, rs3025039, rs3025040), *ACE* (rs1055086, rs4329, rs4461142, rs8066114), *EDN1* (rs2070699, rs2248580, rs5370), SLC64A (rs1042173, rs7224199), *EGLN3* rs1680710, *EPAS1* rs6756667, *HMOX2* rs9921781, *AGT* rs699, *ANGPTL4* rs4076317, and *NOS3* rs1799983 (Supplemental Table S3).

**Table 4:** Analysis of association of SNPs in *PPARA* with cardiac function at high altitude

Gene	SNPs	Crude OR (95% CI)	*p* value	Adjusted OR (95% CI)	*p* value
*PPARA*	rs135538 (G>C)	4.04 (1.57–10.41)	.036	4.09 (1.54–10.87)	.030
	rs4253623 (A>G)	0.91 (0.43–1.93)	.810	1.02 (0.47–2.23)	.960
	rs4253681 (T>C)	0.85 (0.39–1.86)	.680	0.81 (0.36–1.86)	.620
	rs4253747 (T>A)	0.74 (0.33–1.69)	.470	0.73 (0.32–1.69)	.460
	rs6520015 (T>C)	4.73 (2.26–10.21)	<.001	5.26 (2.37–12.11)	<.001
	rs7292407 (C>A)	5.06 (2.35–10.91)	<.001	5.95 (2.61–13.58)	<.001

Abbreviation: SNP, single nucleotide polymorphism; OR, odds ratio; 95% CI, 95% confidence interval; Adjusted for age, current smoking, current drinking, height and weight; *p *<* *.001 indicate statistical significance.

### 3.5 Linkage disequilibrium pattern and haplotype analysis

**Figure 3:**
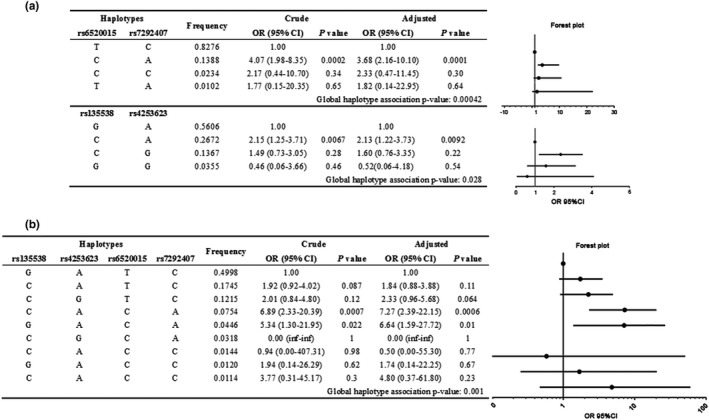
The association of *PPARA* haplotypes and cardiac pumping function at high altitude. (a) The haplotype containing C‐A in rs6520015 and rs7292407 was associated with increased risk for high‐altitude cardiac pumping function reductions (OR: 3.68, 95% CI: 2.16–10.10, *p* = .0001). (b) The haplotype containing C‐A‐C‐A in rs135538, rs4253623, rs650015, and rs7292407 increased the risk for suffering high‐altitude cardiac pumping function reductions with (OR: 7.27, 95% CI: 2.39–22.15, *p *=* *.0006). SNP, single nucleotide polymorphism; OR, odds ratio; 95% CI, 95% confidence interval; Adjusted for age, BMI, current smoking, current drinking; *p *<* *.001 indicates statistical significance

We have shown that three variants in the *PPARA* gene were dangerous in terms of cardiac function reduction during acute high‐altitude exposure. As the loci of rs6520015, rs7292407, and rs135538 were in the same gene, we employed Haploview to evaluate LD in *PPARA*. Supplemental Figure S1 shows the triangular heat map (THM), which indicates the parallel coordinate display by textile plot. The THM inferred two blocks in the SNPs. One block was between rs6520015 and rs7292407, with a D’ value of 0.919 and an R^2^ value of 0.763. The other block was rs4253623 and rs135538, with a D’ value of 0.658 and an *R* ^2^ value of 0.133 (Supplemental Figure S1). The haplotypes of the two blocks that exceeded the cut‐off frequency of >1% are shown in Figure 3a. The haplotypes containing rs6520015 C‐allele and rs7292407 A‐allele were associated with a statistically increased risk for high‐altitude cardiac pumping function reductions. (OR: 3.68, 95% CI: 2.16–10.10, *p *<* *.001) in the adjusted model (Figure 3a). In addition, we further analyzed the haplotypes of the two blocks that included four SNPs. As shown in Figure 3b, the nine most common haplotypes were observed in 99% of the subjects, whereas the others each had frequencies below 1%. The haplotype containing rs135538‐C, rs4253623‐A, rs650015‐C, and rs7292407‐A (C‐A‐C‐A) occupied proportions of 7.54%, and was recognized as significant risk factor (OR: 7.27, 95% CI: 2.39–22.15, *p *<* *.001) adjusted for by age, height, weight, and drinking and smoking history. These data indicated that the individuals who carried the haplotypes C‐A‐C‐A in the *PPARA* gene most likely suffered cardiac function reductions during acute high‐altitude exposure compared with wild‐type carriers.

## 4 DISCUSSION

This self‐controlled study was the first to demonstrate that genetic alterations determine cardiac function changes in acute high‐altitude exposure. We found that the SNPs rs6520015 and rs7292407 in *PPARA* were associated with cardiac pumping function reductions. Moreover, we revealed that carriers of the haplotype C‐A‐C‐A of *PPARA* was at a 7.05‐fold increased risk for suffering cardiac pumping function reductions in acute high‐altitude exposure.

Acute high‐altitude exposure has long been recognized as a cardiac stressor (Naeije, 2010). Most studies have shown that the LV systolic function is well preserved in acute hypoxia as a result of an increase in CO with tachycardia and no change or slight improvement of SV (Bartsch & Gibbs, 2007; De Boeck et al., 2018; Rao et al., 2015). The increase in CO exactly matches the decrease in the arterial oxygen content so that oxygen delivery to tissues remains unchanged. In accordance with previous reports, our study also confirmed that HR, LVEF, and CO increased in most of the subjects. More importantly, we detected a reduction in CO in 48 individuals in the cohort, which suggested that a few lowlanders probably suffered left cardiac pumping function reductions during acute high‐altitude exposure. This reduction in CO has not been shown in previous reports and might be attributed to a slight increase in HR with a significant decrease in SV in the COR group. The HR increase shortened IVRT, reduced LV filling and resulted in a decrease in SV. However, acute hypoxia exerted negative inotropic effects in the myocardium. It has long been thought that the myocardium may self‐limit its pumping function because of decreased oxygen availability (Osculati et al., 2016; Stembridge et al., 2019), which might also account for the reduction in SV and CO as a result of myocardial inadaptation. The adaptation of the myocardium to hypobaric hypoxia depends on myocardial flexibility in utilizing other energy substrates as well as the genetic difference in cardiac metabolism (Kodde, van der Stok, Smolenski, & de Jong, 2007).

The myocardium is able to make use of all varieties of energy substrates, including lipids, ketone bodies, carbohydrates and amino acids, for ATP production in the mitochondria (Kolwicz, Purohit, & Tian, 2013). Among these metabolic pathways, FAO is considered to be the predominant pathway in the adult myocardium. In response to different stimuli, the heart is capable of converting and remodeling metabolic pathways to modulate myocardial energetics and contractile function (Kolwicz et al., 2013). This is associated with complex regulatory mechanisms, including the transcriptional regulation and posttranslational modification of proteins involved in each metabolic pathway. Among the transcriptional regulatory proteins, those involved in the PPARα/PGC1 circuit are regarded as constituting the core mechanism in the adult heart (Finck & Kelly, 2002). PPARα interacts with PGC1 and recruits other coactivators to acetylate histones and initiate the transcription of metabolic genes, such GLUT4, MCPT1 and NRF1(Huss, Levy, & Kelly, 2001; Lehman et al., 2000). When PPARα is conditional knockout in the heart, the expression of genes encoding fatty acid transport mediators and oxidation enzymes is diminished, and the myocardium exhibits a phenotype with fibrosis (Djouadi et al., 1998; Watanabe et al., 2000).

PPARα is encoded by the gene *PPARA*. A previous study has shown that one putatively advantageous haplotype of *PPARA* is related to increased circulating levels of fatty acids in Tibetans (Murray et al., 2018), implying a possible downregulation of FAO. Another study also indicated that the highland natives are capable of utilizing oxygen more efficiently with a lower ratio of FAO in muscle as a result of decreased expression of PPARα (Horscroft et al., 2017). Likewise, *PPARA* is also regulated by HIF via inhibition of the promoter of *PPARA* in hypoxia, which has been reported to be involved in decreasing hemoglobin concentrations in Tibetans (Peng et al., 2011). Although most previous studies have focused on *PPARA* associations with fatty acid metabolism and the hypoxia‐inducible pathway, very little evidence involves the *PPARA* association with cardiac function. In the present study, we determined that the two SNPs and the haplotype C‐A‐C‐A *PPARA* were high‐risk factors for cardiac pumping function reductions during acute high‐altitude exposure, which implied that cardiac FAO was closely related to the variations in cardiac function during acute hypoxia.

The C‐A‐C‐A haplotype comprise the SNPs rs135538, rs4253623, rs6520015, and rs7292407. Both rs135538 and rs4253623 are located at the intron of *PPARA* and exhibit a slight LD in block2 (Supplement Figure S1). However, the G‐A haplotype in block2 shows a nonsignificant association with cardiac function variations. The rs6520015 and rs7292407 loci are beyond the area of the *PPARA* transcript, which reside 90 kb away from the transcription initiation site of *PPARA*. The two SNPs that are formed in tight LD in block1 (*D*’=  0.919 and *R* ^2^ = 0.763) and the C‐allele variation of rs6520015 combined with the A‐allele variation of rs7292407 predominantly increase the risk for cardiac pumping function reductions at high altitude (OR: 3.68, 95% CI: 2.16–10.10). More importantly, once we combined block1 with block2, the risk for cardiac pumping function reductions increased more significantly for the C‐A‐C‐A haplotype (OR: 7.05, 95% CI: 1.25–39.60), suggesting an interaction between block1 and block2. Additionally, in block1, the rs6520015 and rs7292407 loci overlapped in the area of the *MIRLET7B* intron, which encoded miRNA MIRLET7. This area exhibited an extremely high level of lysine 27 acetylation on histone H3 (H3K27AC), which indicated much higher transcriptional activity. Therefore, we speculated that the variation in haplotype might affect miRNA MIRLET7, which further regulates *PPARA* transcription because two SNPs, rs6520015 and rs7292407, are localized in the DNA sequence of *MIRLET7B*. Recent studies have indicated that MIRLET7 regulates the methylation of CDKN2A and NAOGO promoters (Harada‐Shirado et al., 2015; Li et al., 2017), implying a similar possible epigenetic effect on *PPARA*. On the other hand, block1 was beyond the area of the traditional promoters of *PPARA*. Block1 might serve as a remote regulatory element that controls the transcription of *PPARA*, and C‐A might be the major haplotype in regulating the function of remote regulatory elements. However, all possible mechanisms need to be confirmed in future mechanistic research.

In general, although the molecular mechanism of the haplotype that includes four SNPs was unclear until now, the result of the correlation analysis has aroused great interest and supplied important insight into cardiac function variations at high altitude. According to the results above, it is helpful to the clinicians to identify the individuals before departure and prevent their cardiac function from being impaired in acute hypoxia. This study is the first to reveal the role of genetics in cardiac pumping function variations in acute hypoxia and provides a potential predictor for individuals who would most likely suffer cardiac pumping function reductions following acute high‐altitude exposure.

## CONFLICT OF INTEREST

The authors declare no conflict of interest.
