# Supplementary material for: PPARA genetic variants increase the risk for cardiac pumping function reductions following acute high‐altitude exposure: A self‐controlled study
Source: Mol Genet Genomic Med. 2019 Aug 12;7(10):e00919. doi: 10.1002/mgg3.919 (PMC6785441; doi:10.1002/mgg3.919)
Supplement: Supplementary file 1 [file MGG3-7-e00919-s001.docx]

**Supplement table 1.** **Physiological parameters in COR and COI groups at low altitude**

|  | **COR (n = 48)** | **COI (n = 103)** | ***P* value** |
| --- | --- | --- | --- |
| **Demographic parameters** | | | |
| **Age** | 22 (20-26) | 22 (20-25) | 0.686 |
| **Height (cm)** | 171 ± 4 | 172 ± 5 | 0.176 |
| **Weight (kg)** | 63 (60-66) | 64 (60-68) | 0.549 |
| **BMI** | 22 (20-23) | 22 ± 2 | 0.990 |
| **Smoking history** |  |  |  |
| **Yes (n, %)** | 36 (75) | 76 (74) | 0.874 |
| **No (n, %)** | 12 (25) | 27 (26) |  |
| **Drinking history** |  |  |  |
| **Yes (n, %)** | 29 (60) | 65 (63) | 0.751 |
| **No (n, %)** | 19 (40) | 38 (37) |  |
| **Physiological characters** | | | |
| **SpO2 (%)** | 98 (98-99) | 98 (98-99) | 0.731 |
| **SBP (mmHg)** | 116 ± 10 | 117 ± 11 | 0.427 |
| **DBP (mmHg)** | 74 ± 8 | 73 (68-80) | 0.543 |
| **MAP (mmHg)** | 88 ± 8 | 90 ± 10 | 0.462 |

COI: cardiac output improvement group, COR: cardiac output reduction group, BMI: body mass index, SBP: systolic blood pressure, DBP: diastolic blood pressure, MAP: mean arterial pressure, SpO2: oxygen saturation. Values are presented as mean ± standard deviation or median (range interquartile); *P*<0.05 indicate statistical significance.

**Supplement table 2. Genotype frequencies**

| **Gene** | **SNPs** | **Genotypes** | **Frequency cases (n, %)** | **MAF** | ***P*-HWE** |
| --- | --- | --- | --- | --- | --- |
| ***ACE*** | rs1055086 | G/G | 46 (31) | 0.342 | 0.62 |
|  |  | A/G | 71 (47) |  |  |
|  |  | A/A | 33 (22) |  |  |
|  | rs4329 | G/G | 48 (32) | 0.470 | 0.40 |
|  |  | A/G | 78 (52) |  |  |
|  |  | A/A | 23 (15) |  |  |
|  | rs4461142 | C/C | 48 (32) | 0.325 | 0.62 |
|  |  | C/T | 76 (51) |  |  |
|  |  | T/T | 25 (17) |  |  |
|  | rs8066114 | C/C | 100 (67) | 0.293 | 0.60 |
|  |  | C/G | 44 (29) |  |  |
|  |  | G/G | 6 (4) |  |  |
| ***EDN1*** | rs2070699 | T/T | 44 (29) | 0.356 | 1.00 |
|  |  | G/T | 74 (50) |  |  |
|  |  | G/G | 32 (21) |  |  |
|  | rs2248580 | A/A | 54 (36) | 0.389 | 0.86 |
|  |  | A/C | 70 (47) |  |  |
|  |  | C/C | 25 (17) |  |  |
|  | rs5370 | G/G | 80 (54) | 0.247 | 0.68 |
|  |  | G/T | 57 (38) |  |  |
|  |  | T/T | 12 (8) |  |  |
| ***7EGLN1*** | rs12406290 | G/G | 42 (29) | 0.292 | 0.24 |
|  |  | A/G | 80 (55) |  |  |
|  |  | A/A | 24 (16) |  |  |
|  | rs12757362 | G/G | 144 (95) | 0.090 | 1.00 |
|  |  | C/G | 7 (5) |  |  |
|  | rs1339891 | G/G | 125 (83) | 0.185 | 0.14 |
|  |  | A/G | 23 (15) |  |  |
|  |  | A/A | 3 (2) |  |  |
|  | rs1339894 | G/G | 149 (100) | 0.138 | - |
|  | rs1361384 | A/A | 150 (99) | 0.139 | 1.00 |
|  |  | A/G | 1 (1) |  |  |
|  | rs1538667 | A/A | 139 (92) | 0.133 | 0.24 |
|  |  | A/G | 11 (7) |  |  |
|  |  | G/G | 1 (1) |  |  |
|  | rs2009873 | A/A | 40 (27) | 0.492 | 0.19 |
|  |  | A/G | 83 (55) |  |  |
|  |  | G/G | 27 (18) |  |  |
|  | rs2066140 | G/G | 39 (26) | 0.493 | 0.14 |
|  |  | C/G | 84 (56) |  |  |
|  |  | C/C | 27 (18) |  |  |
|  | rs2153364 | G/G | 44 (31) | 0.294 | 0.86 |
|  |  | A/G | 71 (50) |  |  |
|  |  | A/A | 26 (18) |  |  |
|  | rs2275279 | A/A | 72 (48) | 0.117 | 0.57 |
|  |  | A/T | 62 (41) |  |  |
|  |  | T/T | 17 (11) |  |  |
|  | rs2486729 | A/A | 40 (27) | 0.490 | 0.32 |
|  |  | A/G | 81 (54) |  |  |
|  |  | G/G | 28 (19) |  |  |
|  | rs2739513 | A/A | 39 (26) | 0.493 | 0.19 |
|  |  | A/G | 82 (55) |  |  |
|  |  | G/G | 27 (18) |  |  |
|  | rs2808609 | T/T | 139 (92) | 0.133 | 0.24 |
|  |  | C/T | 11 (7) |  |  |
|  |  | C/C | 1 (1) |  |  |
|  | rs508618 | A/A | 125 (83) | 0.200 | 0.60 |
|  |  | A/G | 26 (17) |  |  |
| ***EGLN3*** | rs1680710 | G/G | 143 (95) | 0.113 | 1.00 |
|  |  | A/G | 8 (5) |  |  |
| ***EPAS1*** | rs6756667 | G/G | 101 (66) | 0.277 | 0.12 |
|  |  | A/G | 41 (27) |  |  |
|  |  | A/A | 9 (6) |  |  |
| ***HIF1AN*** | rs10883512 | A/A | 120 (80) | 0.170 | 1.00 |
|  |  | A/G | 29 (19) |  |  |
|  |  | G/G | 1 (1) |  |  |
|  | rs2295778 | C/C | 92 (61) | 0.181 | 0.81 |
|  |  | C/G | 51 (34) |  |  |
|  |  | G/G | 8 (5) |  |  |
| ***HIF1A*** | rs11549467 | G/G | 142 (94) | 0.009 | 1.00 |
|  |  | A/G | 9 (6) |  |  |
|  | rs12434438 | A/A | 80 (54) | 0.422 | 0.83 |
|  |  | A/G | 58 (39) |  |  |
|  |  | G/G | 9 (6) |  |  |
|  | rs2301113 | A/A | 68 (45) | 0.470 | 0.18 |
|  |  | A/C | 72 (48) |  |  |
|  |  | C/C | 10 (7) |  |  |
| ***HMOX2*** | rs9921781 | C/C | 142 (100) | 0.123 | - |
| ***NOS3*** | rs1799983 | G/G | 124 (83) | 0.176 | 0.60 |
|  |  | G/T | 26 (17) |  |  |
| ***PPARA*** | rs4253623 | A/A | 105 (70) | 0.172 | 0.39 |
|  |  | A/G | 40 (26) |  |  |
|  |  | G/G | 6 (4) |  |  |
|  | rs4253681 | T/T | 110 (73) | 0.139 | 0.310 |
|  |  | C/T | 40 (26) |  |  |
|  |  | C/C | 1 (1) |  |  |
|  | rs4253747 | T/T | 114 (76) | 0.126 | 0.470 |
|  |  | A/T | 36 (23) |  |  |
|  |  | A/A | 1 (1) |  |  |
| ***SLC6A4*** | rs1042173 | G/G | 101 (68) | 0.485 | 0.57 |
|  |  | G/T | 41 (28) |  |  |
|  |  | T/T | 6 (4) |  |  |
|  | rs7224199 | T/T | 99 (66) | 0.419 | 1.00 |
|  |  | G/T | 45 (30) |  |  |
|  |  | G/G | 5 (4) |  |  |
| ***VEGFA*** | rs10434 | G/G | 88 (59) | 0.348 | 0.64 |
|  |  | A/G | 55 (37) |  |  |
|  |  | A/A | 6 (4) |  |  |
|  | rs3025039 | C/C | 107 (72) | 0.134 | 0.74 |
|  |  | C/T | 40 (27) |  |  |
|  |  | T/T | 2 (1) |  |  |
|  | rs3025040 | C/C | 110 (73) | 0.151 | 0.74 |
|  |  | C/T | 38 (26) |  |  |
|  |  | T/T | 2 (1) |  |  |
| ***ANGPTL4*** | rs4076317 | G/G | 81 (54) | 0.204 | 0.68 |
|  |  | C/G | 56 (38) |  |  |
|  |  | C/C | 12 (8) |  |  |

SNP, single nucleotide polymorphism; MAF, minor allele frequency; HWE, Hardy-Weinberg Equilibrium

**Supplement table 3. Association of genetic variants and risk on cardiac output reduction**

| **Genes** | **SNPs** | **Crude**  **OR 95%CI** | ***P* value** | **Adjusted**  **OR 95%CI** | ***P* value** |
| --- | --- | --- | --- | --- | --- |
| ***ACE*** | rs1055086 (G > A) | 0.55 (0.27-1.14) | 0.11 | 0.57 (0.27-1.18) | 0.13 |
|  | rs4329 (G > A) | 0.54 (0.26-1.10) | 0.09 | 0.56 (0.27-1.15) | 0.12 |
|  | rs4461142 (C > T) | 0.61 (0.30-1.26) | 0.19 | 0.63 (0.31-1.31) | 0.22 |
|  | rs8066114 (C > G) | 0.91 (0.44-1.90) | 0.80 | 0.91 (0.43-1.92) | 0.81 |
| ***EDN1*** | rs2070699 (T > G) | 1.18 (0.55-2.52) | 0.68 | 1.14 (0.52-2.47) | 0.74 |
|  | rs2248580 (A > C) | 0.92 (0.45-1.88) | 0.83 | 0.95 (0.46-1.98) | 0.90 |
|  | rs5370 (G > T) | 1.41 (0.71-2.81) | 0.33 | 1.40 (0.68-2.86) | 0.36 |
| ***EGLN1*** | rs12406290 (G > A) | 0.97 (0.45-2.08) | 0.94 | 1.03 (0.47-2.23) | 0.95 |
|  | rs12757362 (G > C) | 0.34 (0.04-2.94) | 0.28 | 0.38 (0.04-3.49) | 0.35 |
|  | rs1339891 (G > A) | 0.59 (0.22-1.59) | 0.28 | 0.56 (0.21-1.54) | 0.25 |
|  | rs1339894 (GG) | monomorphic SNP | | | |
|  | rs1361384 (A > G) | 0.00 (0.00-NA) | 0.38 | 0.00 (0.00-NA) | 0.45 |
|  | rs1538667 (A > G) | 0.70 (0.18-2.70) | 0.59 | 0.72 (0.18-2.84) | 0.63 |
|  | rs2009873 (A > G) | 1.89 (0.82-4.38) | 0.12 | 1.89 (0.81-4.40) | 0.13 |
|  | rs2066140 (G > C) | 2.18 (0.92-5.20) | 0.07 | 2.22 (0.92-5.35) | 0.06 |
|  | rs2153364 (G > A) | 0.91 (0.42-1.97) | 0.82 | 0.96 (0.44-2.10) | 0.93 |
|  | rs2275279 (A > T) | 2.09 (0.81-3.24) | 0.16 | 2.17 (0.76-6.24) | 0.15 |
|  | rs2486729 (A > G) | 1.92 (0.83-4.44) | 0.12 | 1.91 (0.82-4.45) | 0.12 |
|  | rs2739513 (A > G) | 1.78 (0.77-4.14) | 0.17 | 1.79 (0.76-4.18) | 0.17 |
|  | rs2808609 (T > C) | 0.70 (0.18-2.70) | 0.59 | 0.72 (0.18-2.84) | 0.63 |
|  | rs508618 (A > G) | 1.43 (0.60-3.44) | 0.43 | 1.49 (0.61-3.66) | 0.38 |
| ***EGLN3*** | rs1680710 (G > A) | 0.70 (0.14-3.62) | 0.67 | 0.76 (0.14-3.99) | 0.74 |
| ***EPAS1*** | rs6756667 (G > A) | 0.66 (0.31-1.41) | 0.28 | 0.64 (0.30-1.39) | 0.25 |
| ***HIF1AN*** | rs10883512 (A > G) | 0.36 (0.13-1.00) | 0.04 | 0.35 (0.12-1.01) | 0.04 |
|  | rs2295778 (C > G) | 0.70 (0.34-1.43) | 0.32 | 0.67 (0.32-1.40) | 0.29 |
| ***HIF1A*** | rs11549467 (G > A) | 0.00 (0.00-NA) | 0.0075 | 0.00 (0.00-NA) | 0.01 |
|  | rs12434438 (A > G) | 1.15 (0.58-2.30) | 0.69 | 1.25 (0.61-2.56) | 0.53 |
|  | rs2301113 (A > C) | 1.41 (0.70-2.83) | 0.33 | 1.42 (0.70-2.87) | 0.33 |
| ***HMOX2*** | rs9921781 (CC) | monomorphic SNP | | | |
| ***NOS3*** | rs1799983 (G > T) | 1.41 (0.59-3.40) | 0.44 | 1.54 (0.63-3.75) | 0.35 |
| ***PPARA*** | rs4253623(A > G) | 0.91 (0.43-1.93) | 0.81 | 1.02 (0.47-2.23) | 0.96 |
|  | rs4253681(T > C) | 0.85 (0.39-1.86) | 0.68 | 0.81 (0.36-1.86) | 0.62 |
|  | rs4253747 (T > A) | 0.74 (0.33-1.69) | 0.47 | 0.73 (0.32-1.69) | 0.46 |
| ***SLC6A4*** | rs1042173 (G > T) | 0.55 (0.25-1.21) | 0.13 | 0.46 (0.20-1.07) | 0.06 |
|  | rs7224199 (T > G) | 0.55 (0.26-1.19) | 0.12 | 0.48 (0.21-1.08) | 0.07 |
| ***VEGFA*** | rs10434 (G > A) | 1.05 (0.52-2.10) | 0.90 | 0.97 (0.47-2.00) | 0.94 |
|  | rs3025039 (C > T) | 1.44 (0.68-3.05) | 0.21 | 1.54 (0.72-3.31) | 0.27 |
|  | rs3025040 (C > T) | 0.88 (0.40-1.93) | 0.75 | 0.94 (0.42-2.10) | 0.89 |
| ***ANGPTL4*** | rs4076317 (G > C) | 0.54 (0.26-1.09) | 0.08 | 0.58 (0.28-1.21) | 0.14 |

SNP: single nucleotide polymorphism; OR: odds ratio; 95% CI: 95% confidence interval; Adjusted for age, current smoking, current drinking, height and weight; *P*<0.001 indicates statistical significance.

**Supplement Figure 1. Haplotype block map for the SNPs of *PPARA***

**
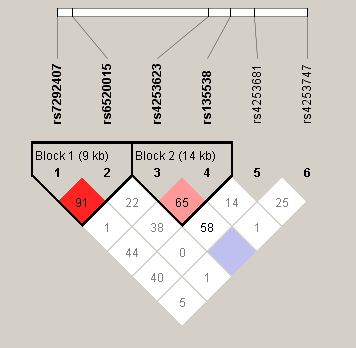
**

| **SNPs** | **Blocks** | **D’** | **R^2^** | **LOD** |
| --- | --- | --- | --- | --- |
| **rs7292407 and rs6520015** | **Block1** | 0.919 | 0.763 | 29.51 |
| **rs4253623 and rs135538** | **Block2** | 0.658 | 0.133 | 5.15 |

Linkage disequilibrium plot constructed by Haploview 4.2 software

**Supplement table 4. Primer Sequences**

| **SNP Name** | **Primer** | **Sequence (5' to 3')** |
| --- | --- | --- |
| rs4076317 | rs4076317_W1_1F | ACGTTGGATGACCCCGCCTCCAAGACTCCT |
| rs11549467 | rs11549467_W1_2F | ACGTTGGATGTTGAGGACTTGCGCTTTCAG |
| rs11816840 | rs11816840_W1_3F | ACGTTGGATGCAAACACATAGGCTTGCGTC |
| rs12097901 | rs12097901_W1_4F | ACGTTGGATGAAAAGTAAAGGCCAAGCCCC |
| rs3025040 | rs3025040_W1_5F | ACGTTGGATGAGACAGATCACAGGTACAGG |
| rs1339894 | rs1339894_W1_6F | ACGTTGGATGGCTGTTATGTGCCCAATCTG |
| rs1051308 | rs1051308_W1_7F | ACGTTGGATGTTGAGTGCTGGAGCATTTAC |
| rs11190613 | rs11190613_W1_8F | ACGTTGGATGAGAAGGTTTCAGGTCTGAGC |
| rs2301113 | rs2301113_W1_9F | ACGTTGGATGCCTTTGAACTGAGAAGGCAC |
| rs2808609 | rs2808609_W1_10F | ACGTTGGATGTGGGCTTCCCTAACATGACA |
| rs5370W1 | rs5370_W1_11F | ACGTTGGATGTTGTGGGTCACATAACGCTC |
| rs1042173 | rs1042173_W1_12F | ACGTTGGATGAGGTTCTAGTAGATTCCAGC |
| rs3813034 | rs3813034_W1_13F | ACGTTGGATGTCCATACACAATTGAGTTGG |
| rs6726395 | rs6726395_W1_14F | ACGTTGGATGTCACGTGCCTCATTTGATCC |
| rs2153364 | rs2153364_W1_15F | ACGTTGGATGCCATCAAATAGAACACTTGC |
| rs7224199 | rs7224199_W1_16F | ACGTTGGATGATCCCTCACTGACCTAACTC |
| rs11292W1 | rs11292_W1_17F | ACGTTGGATGGCACTGTGCTAAAGCTTTAC |
| rs11156819 | rs11156819_W1_18F | ACGTTGGATGTCTCTGTGGCATCTACCACC |
| rs2486729 | rs2486729_W1_19F | ACGTTGGATGAAAAGGGTCTGCCCTTTTCC |
| rs1054399 | rs1054399_W1_20F | ACGTTGGATGATCCCTGATCATCTTAGGGC |
| rs12406290 | rs12406290_W1_21F | ACGTTGGATGTCAAAAGCAGGATATAAGG |
| rs2739513 | rs2739513_W1_22F | ACGTTGGATGCTTAAATGTGTATATAGCTC |
| rs12757362 | rs12757362_W1_23F | ACGTTGGATGGGTAGTTGCCGTGTATTTCC |
| rs1680710 | rs1680710_W1_24F | ACGTTGGATGCAGACGAGAACTCAAATAGC |
| rs4076317 | rs4076317_W1_1R | ACGTTGGATGGCCCGAGGACGGTTTTTATA |
| rs11549467 | rs11549467_W1_2R | ACGTTGGATGCTTCCAGTTACGTTCCTTCG |
| rs11816840 | rs11816840_W1_3R | ACGTTGGATGCCTGACTATTGAGGGTGTTG |
| rs12097901 | rs12097901_W1_4R | ACGTTGGATGTTCGGCAGCCACCGCCGAG |
| rs3025040 | rs3025040_W1_5R | ACGTTGGATGACAGCAATGTCCTGAAGCTC |
| rs1339894 | rs1339894_W1_6R | ACGTTGGATGGTACCCACTTCATAGCATTG |
| rs1051308 | rs1051308_W1_7R | ACGTTGGATGTCACCCAGAATCCCTCTAAC |
| rs11190613 | rs11190613_W1_8R | ACGTTGGATGGTGAGGAGACAACAGCTTCT |
| rs2301113 | rs2301113_W1_9R | ACGTTGGATGTTAGGTTTCTACTCCCACCC |
| rs2808609 | rs2808609_W1_10R | ACGTTGGATGCTTGCCAGCAGACACTATTG |
| rs5370W1 | rs5370_W1_11R | ACGTTGGATGATTAGGTCGGAGACCATGAG |
| rs1042173 | rs1042173_W1_12R | ACGTTGGATGGAGAGAACAGGGATGCTATC |
| rs3813034 | rs3813034_W1_13R | ACGTTGGATGGGGCCCATTACAAATATATG |
| rs6726395 | rs6726395_W1_14R | ACGTTGGATGGATAATATTTGAGGGGTTTGG |
| rs2153364 | rs2153364_W1_15R | ACGTTGGATGCCTGAAATCAAGGATCTTTGG |
| rs7224199 | rs7224199_W1_16R | ACGTTGGATGCTTCTTTCAGCATGCTAGAC |
| rs11292W1 | rs11292_W1_17R | ACGTTGGATGATAATAACATGGGTGGAGGG |
| rs11156819 | rs11156819_W1_18R | ACGTTGGATGAGCGAGGGAATGAACCTTAC |
| rs2486729 | rs2486729_W1_19R | ACGTTGGATGCCAATATATACCACGCTACC |
| rs1054399 | rs1054399_W1_20R | ACGTTGGATGCAGACACAAAACAAGAGGAC |
| rs12406290 | rs12406290_W1_21R | ACGTTGGATGCTGTATATTTGATGAAGAGG |
| rs2739513 | rs2739513_W1_22R | ACGTTGGATGCATAAAACATTGCCAAACTTC |
| rs12757362 | rs12757362_W1_23R | ACGTTGGATGCCCATGAGCAATATTTCGTC |
| rs1680710 | rs1680710_W1_24R | ACGTTGGATGATGAGCTCACCCTTTAACAC |
| rs4076317 | rs4076317_W1_1U | CTCCTCCGCCCACTC |
| rs11549467 | rs11549467_W1_2U | CGCTTTCAGGGCTTG |
| rs11816840 | rs11816840_W1_3U | GCCTCTGCCAGACCCC |
| rs12097901 | rs12097901_W1_4U | ACGGCCGCGTCGCCGT |
| rs3025040 | rs3025040_W1_5U | ACAGGGATGAGGACAC |
| rs1339894 | rs1339894_W1_6U | GCCCAATCTGTGTTCTAA |
| rs1051308 | rs1051308_W1_7U | TGGAGCATTTACACAGCG |
| rs11190613 | rs11190613_W1_8U | GCCGAATGAGTCCTTATTA |
| rs2301113 | rs2301113_W1_9U | GGGGTAGAAGGCACACTCCT |
| rs2808609 | rs2808609_W1_10U | AACATGACAGCTTTCTTCAAT |
| rs5370W1 | rs5370_W1_11U | GGAAGAACGCTCTCTGGAGGG |
| rs1042173 | rs1042173_W1_12U | AGTAGATTCCAGCAATAAAATT |
| rs3813034 | rs3813034_W1_13U | GTTGAGTTGGTAGAATTTGTTA |
| rs6726395 | rs6726395_W1_14U | CCTGTGATCCACTAACATCTGTA |
| rs2153364 | rs2153364_W1_15U | CCTTGCAACACTTTAATGAATAC |
| rs7224199 | rs7224199_W1_16U | TAACTCCATTATCTTCACTCTTTC |
| rs11292W1 | rs11292_W1_17U | TGTGCTAAAGCTTTACAAACATTG |
| rs11156819 | rs11156819_W1_18U | CCACCTCCATTTTTTTCTTTTCAAA |
| rs2486729 | rs2486729_W1_19U | CTTCGTTTTTCTCCTTAACCATTTC |
| rs1054399 | rs1054399_W1_20U | CCTCTTTGACAGCTCTGTTTATTTTG |
| rs12406290 | rs12406290_W1_21U | TAAGGAATTATTGTAAACCATATTGT |
| rs2739513 | rs2739513_W1_22U | ATGTATATAGCTCACATTTATTTCAAG |
| rs12757362 | rs12757362_W1_23U | GGAGAACATCATGAGGCACATATGTCT |
| rs1680710 | rs1680710_W1_24U | TCCAATTCCTTTTTTAAAATACATCACC |
| rs7542797 | rs7542797_W2_1F | ACGTTGGATGAAAGGGATACAACCACCTGC |
| rs10434W2 | rs10434_W2_2F | ACGTTGGATGATGGGCTGCTTCTTCCAACA |
| rs508618W | rs508618_W2_3F | ACGTTGGATGATCTTTTGGCCTTGAGATCC |
| rs25648W2 | rs25648_W2_4F | ACGTTGGATGGAAGAGTAGCTCGCCGAGG |
| rs1538667 | rs1538667_W2_5F | ACGTTGGATGTTTGATCTCGAACTCCTCGG |
| rs2009873 | rs2009873_W2_6F | ACGTTGGATGGTTTGGATTCTCAGCACTTG |
| rs1055086 | rs1055086_W2_7F | ACGTTGGATGCTTGACGTTGATATAAAGGGC |
| rs12097901 | rs12097901_W2_8F | ACGTTGGATGTGGAACAGCGATGAGCGGG |
| rs3750633 | rs3750633_W2_9F | ACGTTGGATGCCCAGCTTTTGGTTGTCATC |
| rs1361384 | rs1361384_W2_10F | ACGTTGGATGGCCTGAATAGGTGAAAATCC |
| rs2739513 | rs2739513_W2_11F | ACGTTGGATGCATAAAACATTGCCAAACTTC |
| rs8081028 | rs8081028_W2_12F | ACGTTGGATGTCTCTGTCTGTTCCGCATAG |
| rs12406290 | rs12406290_W2_13F | ACGTTGGATGTCAAAAGCAGGATATAAGG |
| rs7542797 | rs7542797_W2_1R | ACGTTGGATGGCAGAGGGAGACTTCAATTC |
| rs10434W2 | rs10434_W2_2R | ACGTTGGATGTCTCACCTGCTTCTGAGTTG |
| rs508618W | rs508618_W2_3R | ACGTTGGATGGTCTATAGCAAGGGTATTTG |
| rs25648W2 | rs25648_W2_4R | ACGTTGGATGCCAAGACAGCAGAAAGTTCA |
| rs1538667 | rs1538667_W2_5R | ACGTTGGATGTAACACTACTCACTTCACAG |
| rs2009873 | rs2009873_W2_6R | ACGTTGGATGGGTGATACAGATCTGTCAAC |
| rs1055086 | rs1055086_W2_7R | ACGTTGGATGCTCCTCACTCCCTGCATTG |
| rs12097901 | rs12097901_W2_8R | ACGTTGGATGAAAAGTAAAGGCCAAGCCCC |
| rs3750633 | rs3750633_W2_9R | ACGTTGGATGAGATACATACATGCGTGTGC |
| rs1361384 | rs1361384_W2_10R | ACGTTGGATGCCATCAAATAGAACACTTGC |
| rs2739513 | rs2739513_W2_11R | ACGTTGGATGCCTAAAACTGGACTAATGGC |
| rs8081028 | rs8081028_W2_12R | ACGTTGGATGTAAAATCCACCTACCTACTC |
| rs12406290 | rs12406290_W2_13R | ACGTTGGATGGCCATTTACGCATGCAGCAG |
| rs7542797 | rs7542797_W2_1U | CTGCCTACTGCTGTG |
| rs10434W2 | rs10434_W2_2U | GACATCTGCCAGTGG |
| rs508618W | rs508618_W2_3U | GCCCCTCATTGGCTTCT |
| rs25648W2 | rs25648_W2_4U | GGCCCCGGTCGGGCCTC |
| rs1538667 | rs1538667_W2_5U | CTGTGGTTACAGACTTGA |
| rs2009873 | rs2009873_W2_6U | TTGAATTCTTGCCCTTTTA |
| rs1055086 | rs1055086_W2_7U | AAAGTCATAAAAATGTCCCT |
| rs12097901 | rs12097901_W2_8U | CCTACGCCGGCGGCCGCACGA |
| rs3750633 | rs3750633_W2_9U | TGTATGTTAGTCTGTCAACTTC |
| rs1361384 | rs1361384_W2_10U | GGAACTATTTGAAATCAGTATTT |
| rs2739513 | rs2739513_W2_11U | TCCCAAAACACTTCTAATGGTAAA |
| rs8081028 | rs8081028_W2_12U | ACCTATTTATCAATACTATGATGGG |
| rs12406290 | rs12406290_W2_13U | GAAGGAATTATTGTAAACCATATTGT |
